# Cellular forces and matrix assembly coordinate fibrous tissue repair

**DOI:** 10.1038/ncomms11036

**Published:** 2016-03-16

**Authors:** Mahmut Selman Sakar, Jeroen Eyckmans, Roel Pieters, Daniel Eberli, Bradley J. Nelson, Christopher S. Chen

**Affiliations:** 1 Institute of Robotics and Intelligent Systems, Eidgenössische Technische Hochschule Zürich, 8092 Zurich, Switzerland; 2 Department of Biomedical Engineering, Boston University, 36 Cummington Mall, Boston, Massachusetts 02215, USA; 3 Wyss Institute for Biologically Inspired Engineering, Harvard University, Boston, Massachusetts 02115, USA; 4 Laboratory for Urologic Tissue Engineering and Stem Cell Therapy, Department of Urology, University Hospital, Zurich 8091, Switzerland

## Abstract

Planar *in vitro* models have been invaluable tools to identify the mechanical basis of wound closure. Although these models may recapitulate closure dynamics of epithelial cell sheets, they fail to capture how a wounded fibrous tissue rebuilds its 3D architecture. Here we develop a 3D biomimetic model for soft tissue repair and demonstrate that fibroblasts ensconced in a collagen matrix rapidly close microsurgically induced defects within 24 h. Traction force microscopy and time-lapse imaging reveal that closure of gaps begins with contractility-mediated whole-tissue deformations. Subsequently, tangentially migrating fibroblasts along the wound edge tow and assemble a progressively thickening fibronectin template inside the gap that provide the substrate for cells to complete closure. Unlike previously reported mechanisms based on lamellipodial protrusions and purse-string contraction, our data reveal a mode of stromal closure in which coordination of tissue-scale deformations, matrix assembly and cell migration act together to restore 3D tissue architecture.

## Abstract (web summary)

Planar *in vitro* models for wound closure stress the role of lamellipodial protrusions and purse-string contraction. Here the authors develop a 3D biomimetic model for tissue repair and show a mode of stromal closure that relies on whole tissue deformations, cell migration and matrix deposition.

## Graphical abstract

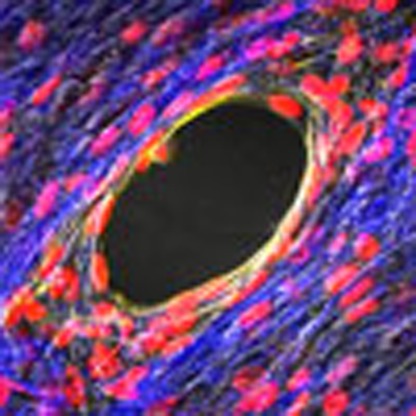

Closure of open gaps within a tissue, a morphogenetic process involving rearrangement of cells and assembly of extracellular matrix (ECM), is fundamental for normal development and repair of damaged tissues and organs^1^,^2^,^3^. During embryogenesis, many tissue structures such as the neural tube^4^,^5^, eyelid^6^ and dorsal epidermis^7^,^8^ require closure as a key step in forming a contiguous anatomical structure. In adults, tissue closure is again invoked during the wound-healing response after injury, to restore mechanical integrity and function^9^,^10^,^11^.

The mechanical basis of wound healing has been studied using *in vitro* models that measure cellular forces during epithelial gap closure through collective cell migration on planar substrates^12^,^13^,^14^,^15^,^16^,^17^,^18^. These studies revealed one class of closure that involves covering a bare surface in which leading cells in an advancing epithelial monolayer migrate across the surface in an adhesion-dependent manner^14^,^15^,^16^. Across non-adhesive gaps, epithelial cells employ a different mechanism by generating traction forces parallel to the wound margin through the contraction of a multicellular actin purse string to close the gap^17^,^18^. Although these mechanisms explain many aspects of re-epithelialization, it is unclear how these findings relate to repair of fibrous tissues wherein mesenchymal cells ensconced in a fibrillar matrix restore the three-dimensional (3D) architecture of the tissue.

Here we introduce a 3D bioengineered culture system to study the cellular and biophysical processes during stromal gap closure. We generated arrays of 3D microscale tissues (microtissues) consisting of 3T3 fibroblasts embedded in a type I collagen matrix^19^. The microtissues were suspended between flexible cantilevers that simultaneously constrain the microtissues and report microtissue tension in real time. After formation, microtissues were damaged with a microsurgical knife mounted to a microrobotic manipulation platform and closure was monitored using time-lapse confocal microscopy. We demonstrate that, in contrast to mechanisms previously described by planar *in vitro* models, fibroblasts close the open gap through the coordinated action of force-dependent contraction of the whole tissue, circumferential cell migration around the wound edge and assembly of fibronectin scaffolding that allows cells to repair the gap and restore tissue integrity.

## Results

### Fibroblasts repair gaps in 3D collagen microtissues

**Figure 1:**
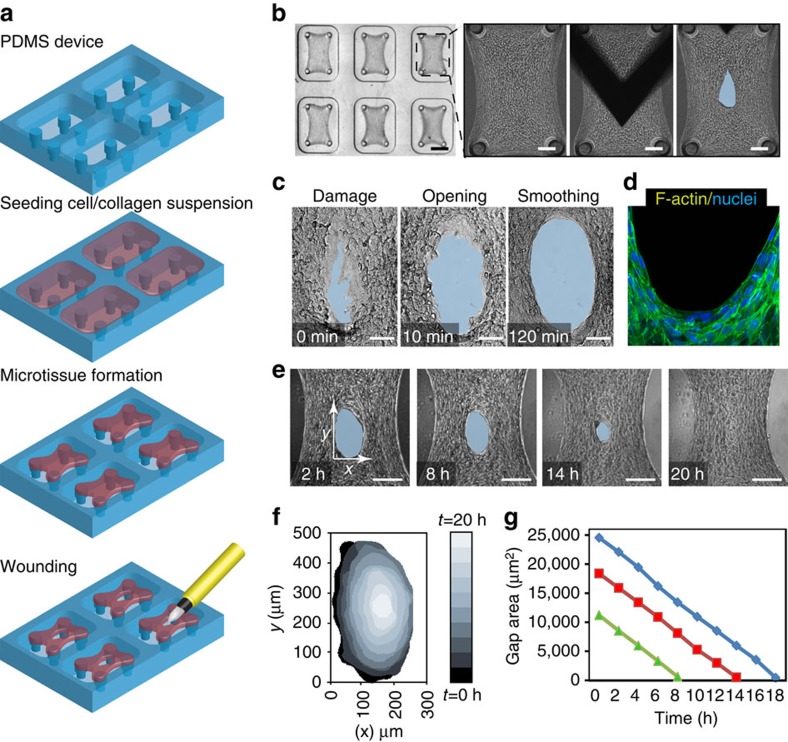
Wounding and closure of 3D microtissues. (**a**) Schematic of microtissue device and workflow. 3T3 fibroblasts are seeded in a collagen type I suspension, and after tissue formation microtissues are damaged using a microdissection knife. (**b**) Micrographs of microtissues before, during and after damage. The blue zone demarcates the gap area (original magnification × 2.5 and scale bar, 500 μm; and × 10 and scale bar, 100 μm). (**c**) Temporal sequence of wound area showing opening and smoothing of the wound edge (original magnification × 40and scale bar, 50 μm). (**d**) Staining for F-actin (Phalloidin, green) and nuclei (Hoechst, blue) showing alignment of cells at the wound edge (original magnification × 63 and scale bar, 50 μm). (**e**) Temporal sequence of micrographs showing closure (original magnification × 15 and scale bar, 100 μm). (**f**) Visual representation of spatiotemporal dynamics of gap area during closure. (**g**) Graph showing gap area in function of time for three tissues with different initial wound size (each colour represents one gap).

To generate microtissues, we seeded NIH-3T3 fibroblasts in a suspension of collagen type I into microfabricated substrates with arrays of wells containing vertical cantilevers (Fig. 1a and Supplementary Fig. 1). Cells contracted the collagen to form a dense fibrocellular microtissue around the caps of the engineered cantilevers in each well. To examine the response of these microtissues to damage, we wounded them in the centre of the tissue with a microdissection knife mounted on a teleoperated micromanipulator and then observed how they evolved (Fig. 1b). Within minutes after the full-thickness incision was made, the gap further widened (Fig. 1c). As the area of the gap stabilized over the following several hours, the rough wound edge smoothened to form an ellipse (Fig. 1c), a process associated with alignment and elongation of the cells along the circumferential boundary of the wound edge (Fig. 1d). Over the course of the next 24 h, the gap progressively closed, while maintaining its elliptical shape and keeping the centroid position of the wound stationary (Fig. 1e and Supplementary Movie 1). Temporal analysis of the gap area (Fig. 1f) showed a constant rate of closure (1,344±150 μm^2^ h^−1^) throughout the process independent of the initial gap size (Fig. 1g), until the gap closed.

### Closure is driven by contractility rather than proliferation

During wound healing, cell proliferation is necessary to populate the wound with ample matrix-producing fibroblasts^20^. To test whether cell proliferation and resulting tissue expansion could contribute to the filling of the gap, we administered the nucleotide analogue 5-ehynyl-2′-deoxyuridine (EdU) to label fibroblasts undergoing proliferation during closure and treated microtissues with Aphidicolin, a mitogenic inhibitor. EdU uptake was substantial over the 24 h of wound closure; however, whereas aphidicolin nearly eliminated EdU uptake by the cells, microtissue gap closure remained intact (Supplementary Fig. 2 and Supplementary Movie 2).

**Figure 2:**
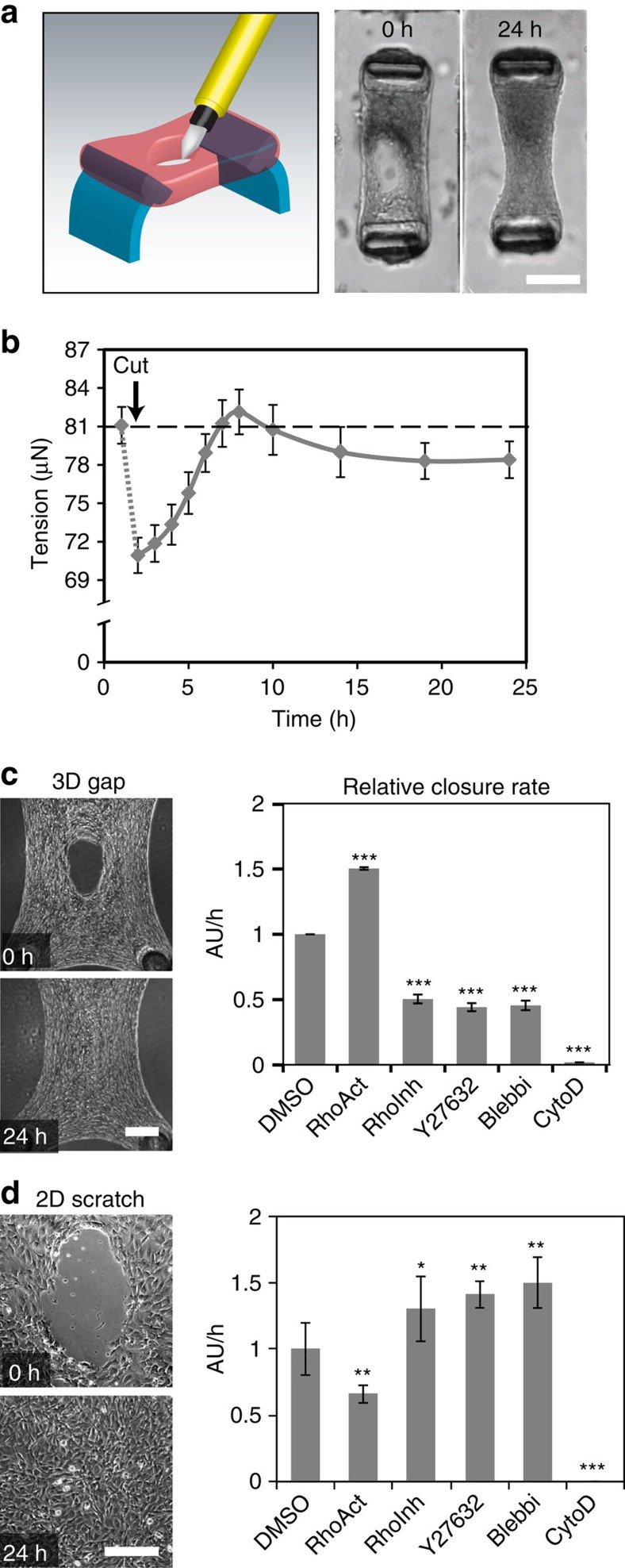
Gap closure is mediated by cytoskeletal contractility. (**a**) Healing response occurs in microtissues anchored to two cantilevers, allowing measuring contractility during gap closure. (**b**) Time course of microtissue tension generated during healing. Each dot represents the average tension for five microtissues±s.e.m. Dashed line represents the baseline tension before microtissue damaging. (**c**) Relative closure rate measured at 10 h after damage in the presence of RhoActivator II (RhoAct), RhoInhibitor (RhoInhib), Y27632, Blebbistatin (Blebbi) and Cytochalasin D (CytoD) (*N*=6 to 12 microtissues per experiment, 3 experiments). (**d**) Closure rate of 2D elliptical scratch wounds in the presence of cytoskeletal drugs (*N*=3 per experiment, 3 experiments). All bar graphs represent average ±s.e.m. Statistical analysis: analysis of variance (ANOVA), *post-hoc* Dunnett's test for comparison with the dimethyl sulfoxide (DMSO) control, *P≤0.05, **P≤0.01 and ***P<0.001. Scale bar, 100 μm (in all images).

Cell-generated tension has previously been shown to be critical to closure of gaps^16^. To investigate this possibility in our model, we monitored microtissue tension across two deformable cantilevers during repair (Fig. 2a). We observed the baseline tissue tension rapidly decline after damage (coincident with the initial gap widening), followed by a progressive increase that peaked at 10 h, and settled at a steady state consistently close to but slightly below the baseline tension (Fig. 2b). The dynamic mechanical response triggered by wounding suggests a potential role for cell-generated forces in gap closure. We targeted the small G-protein Rho, a central regulator of actin dynamics and cytoskeletal contractility. Activation of Rho signalling with a cell-permeable form of cytotoxic necrotizing factor catalytic domain-derived protein increased the closure rate, whereas Rho inhibition with a cell-permeable C3 transferase decreased closure (Fig. 2c). We next examined whether the effects of Rho signalling on wound closure resulted from its effects on actin filament re-organization or myosin II activity. As expected, disruption of the actin cytoskeleton with Cytochalasin D completely abolished the closure response. Inhibition of either non-muscle myosin II activity with Blebbistatin or Rho kinase activity with Y-27632 also significantly decreased the closure rate, similar to the effects of inhibiting Rho signalling. In sharp contrast to these findings, activation of Rho signalling in the two-dimensional (2D) wound assays slowed down closure, whereas inhibition of Rho signalling or contractility accelerated closure, whether the closure occurred across an elliptical gap (Fig. 2d) or a classical parallel scratch wound (Supplementary Fig. 3). Taken together, these data indicate that cellular contractility is required for closure of the gaps in our 3D model, and that the mechanism for closure appears to be different in 2D versus 3D gaps (Supplementary Movie 3).

### Contractility controls migration along the wound edge

**Figure 3:**
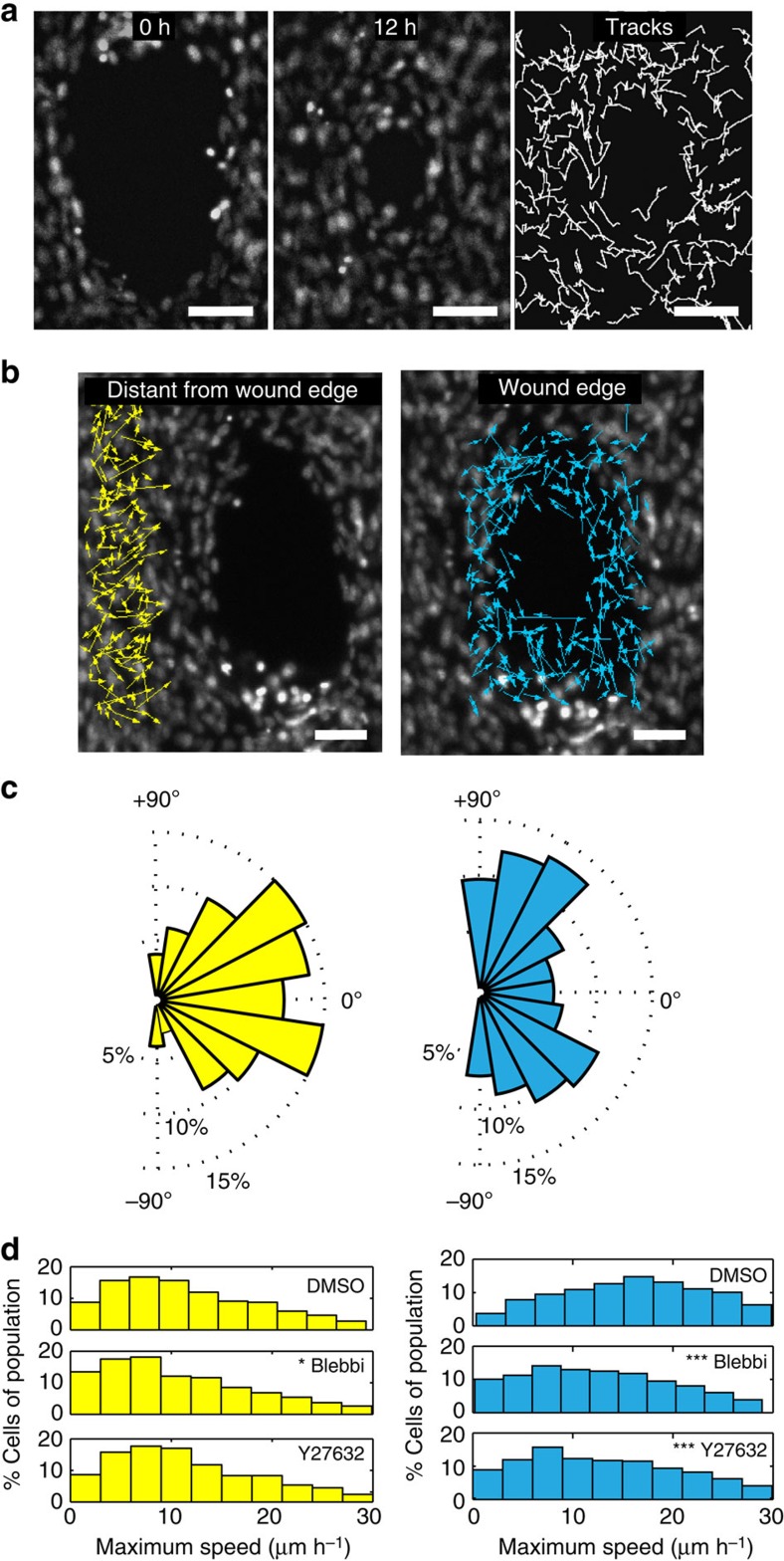
Gap closure is mediated by cell migration. (**a**) Two frames of Hoechst labelled 3T3 fibroblasts in a wounded microtissue taken at 0 and 12 h of a time-lapse sequence during closure. Intermittent tracks of nuclei show non-correlated migration between neighbouring cells. (**b**) Vector fields showing net displacements for cells located at the wound edge versus 100 μm distant of the wound edge during closure. (**c**) Windrose plots displaying the frequency of migrating cells (%) versus the radial migration angle (0° means displacement towards the gap, 90° means tangential movement). (**d**) Histograms showing the maximum speed of cells distant of the wound edge when treated with dimethyl sulfoxide (DMSO), Blebbistatin and Y27632 (*N*=700–1,000 cells from 4 microtissues per condition, Kruskal–Wallis test, *post-hoc* test: Dunn Method For Joint Ranking with DMSO condition as control, *P<0.05 and ***P<0.001). Scale bars, 100 μm.

The contractility requirement for closure and the alignment of cells along the wound edge suggested the possibility that the fibroblasts adopted an actomyosin purse string, as has been described as a key element to gap closure by the epithelium^21^,^22^. However, immunofluorescence imaging of phosphorylated myosin light chain was characterized by discontinuous, punctate distributions inconsistent with purse strings (Supplementary Fig. 4). Furthermore, time-lapse movies of cells (Supplementary Movie 4) showed that neighbouring fibroblasts followed non-correlated trajectories, especially around the wound edge (Fig. 3a), which are distinct from the collective motions described for epithelial sheets^13^,^23^. Cells at the wound edge migrated tangentially in both directions along the circumference of the wound, whereas cells located distal from the wound edge generally moved radially towards the centre of the gap (Fig. 3b,c). As cell-generated forces drive gap closure (Fig. 2), we hypothesized that this could be mediated via cell migration. Indeed, treatment of microtissues with Y27632 or Blebbistatin reduced more the maximum migration speeds of cells (Fig. 3d) located at the wound edge than those distal from the wound edge.

### Fibroblasts tow fibronectin into the gap area

**Figure 4:**
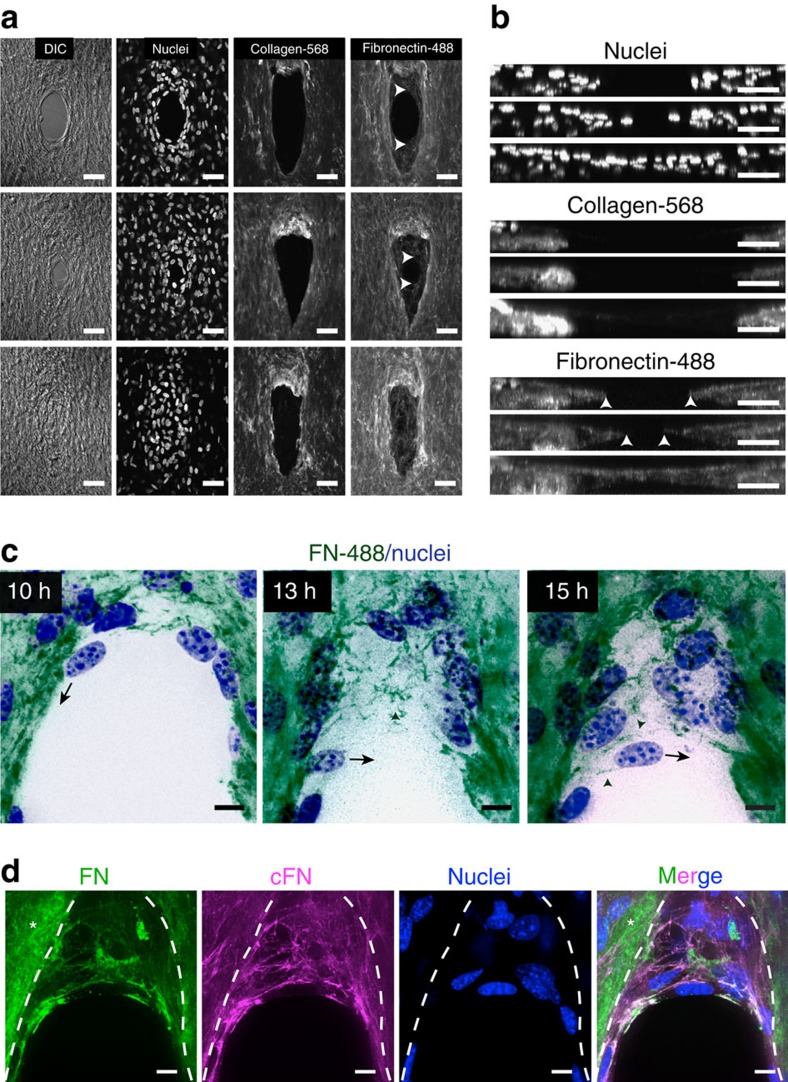
Stromal gap closure requires deposition of a provisional fibrous matrix. (**a**) Confocal micrographs of closing microtissues at three different stages of closure. Tissues were stained with Hoechst (nuclei), fluorescently labelled collagen (Collagen-568) and fluorescently labelled fibronectin (Fibronectin-488). White arrows depict fibronectin at the wound edge (original magnification × 40). (**b**) Cross-sectional view along *y* axis of the same microtissues (scale bar, 100 μm). (**c**) Time-lapse sequence showing fibronectin scaffolding in the gap area. Black arrow indicates migration direction of the same cell at different time points during closure. Green arrowheads depict fibronectin fibres forming a substrate for cell migration into the gap area. (Green: Alexa-488-labelled fibronectin, blue: nuclei, original magnification × 63 and scale bar, 25 μm). (**d**) Antibody staining for total fibronectin (green) and cellular fibronectin (magenta). Nuclei (blue) are stained with Hoechst (original magnification × 63 and scale bar, 25 μm).

The non-correlated movements of the fibroblasts suggested that cells primarily coordinate with the ECM rather than via cell–cell adhesions, to mediate the gap closure response. Indeed, knockdown of N-cadherin in 3T3 fibroblasts did not impede gap closure (Supplementary Fig. 5). This finding led us to investigate how the ECM evolved during repair. The microtissue matrix consists mainly of type I collagen; thus, we asked whether closure was primarily driven by the contraction of the existing collagen matrix, or the process involved assembly of new matrix inside the gap. Time-lapse microscopy of gap closure in microtissues containing fluorescently tagged collagen type I matrix showed that reduction of gap area for the first 10 h was primarily associated with contraction of the hole within the existing collagen matrix (Supplementary Movie 5). Interestingly, a later stage of closure followed in which the cells continued to enter and fill the gap area without any collagen matrix (Fig. 4a and Supplementary Movie 5).

The lack of type I collagen within the gap suggested that cells employed a second, more provisional matrix for later stages of wound closure. Indeed, fluorescently labelled fibronectin spiked into the medium showed that fibronectin was being deposited into the gap throughout the closure (Fig. 4a,b). High-resolution time-lapse microscopy revealed that fibroblasts first migrated circumferentially around the wound edge, while towing, remodelling and extending existing fibronectin fibres into the gap (Fig. 4c and Supplementary Movie 6). Furthermore, fibroblasts also deposited cellular fibronectin during this remodelling process (Fig. 4d). The newly formed fibrillar fibronectin template then served as a substrate for cells to migrate further into the gap area (Fig. 4c). During the final phases of healing, a single layer of cells then fully closed the gap with this fibronectin, and then this layer thickened and reinforced as additional cells entered the region, ultimately resulting in a multilayered, matrix-rich tissue.

### Cell-matrix adhesion governs provisional matrix assembly

**Figure 5:**
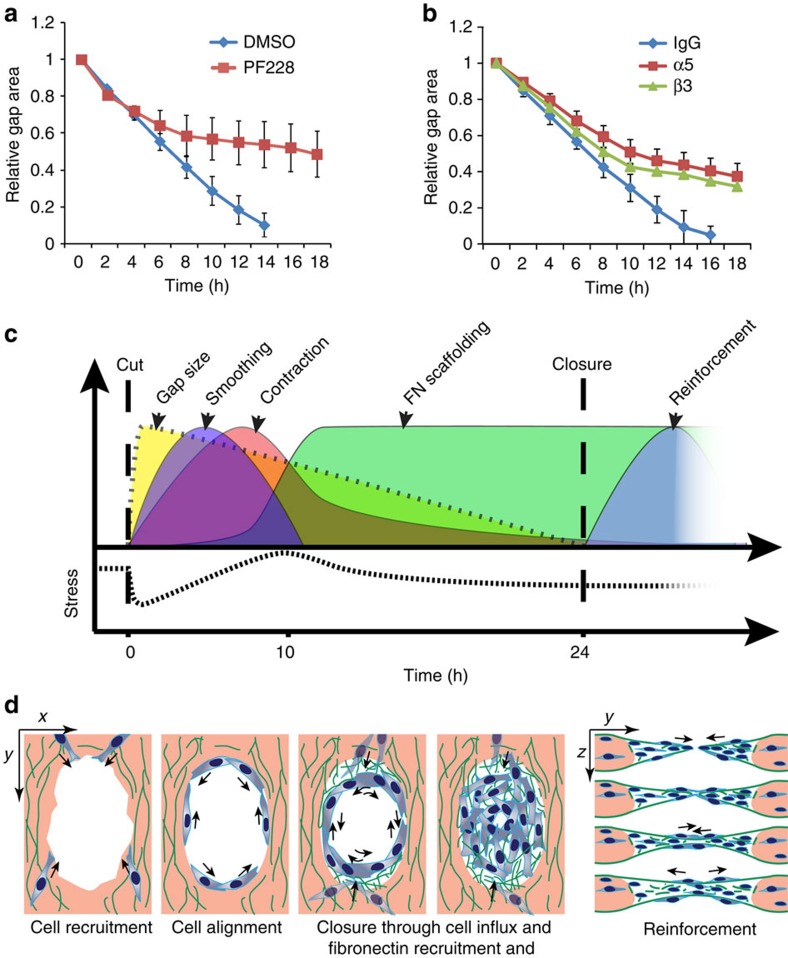
Matrix assembly depends on FAK signalling and integrin binding. (**a**) Relative gap closure in the presence of the FAK inhibitor PF228. Dimethyl sulfoxide (DMSO) is vehicle control (*N*=9 tissues per condition, 3 experiments). (**b**) Relative gap closure in the presence of α5 and β3 antibodies (*N*=14 tissues per condition, 3 experiments). Rat IgG serves as antibody isotype control. Error bars represent average ±s.e.m. (**c**) Graphical illustration of different stages of gap closure. (**d**) Schematic drawing illustrating gap closure through tangential cell movement and deposition of a provisional fibronectin template for cell entry and closure. On closure, continuous cell migration and fibronectin remodelling reinforces the closure area vertically.

Remodelling of ECM is regulated by the binding of matrix proteins to cells through integrin receptors. Once bound to ligands, integrins cluster and activate focal adhesion kinase (FAK), leading to assembly of focal adhesions and modulation of Rho GTPases^24^,^25^. In our model, inhibition of FAK kinase activity using the small compound PF-228 abolished closure (Fig. 5a). Interestingly, despite FAK is activated by most integrins^26^, PF-228 primarily hindered closure 8–10 h after injury, which coincided with the onset of fibronectin scaffolding. To further elucidate the role of integrins and cell–matrix adhesion in stromal wound closure, we treated tissues with blocking antibodies that affect interactions with fibronectin. Using α5- or β3-integrin antibodies targeting α5β1 and αVβ3, we observed a small decrease in closure rate, during the collagen contraction phase of gap closure, and a more pronounced inhibition of the fibronectin-dependent phase of closure (Fig. 5b). These findings are consistent with a more critical role for fibronectin in the later stages of gap closure.

## Discussion

Cells employ different mechanisms to close gaps during morphogenesis. During dorsal closure, epithelial cells assemble a contractile multicellular actin structure that delineates the wound edge and acts as a purse string^27^,^28^. Through coordinated actions of pulling on the actin ring and altering the shape of the cells surrounding the wound, the gap area narrows. Once the opposing epithelial fronts are within filopodial reach of one another, cells ‘zip' the wound edges together^11^. A similar zippering mechanism is used by neural ectodermal cells to close the hindbrain and the spinal cord in mammals^29^. In contrast, closure of the midbrain involves the formation of flexible, motile cell extensions and cellular bridges between the closing folds^5^. In our model, we did not see evidence for these previously described closure mechanisms. Instead, gap closure for stromal tissue appeared to be a staged process comprising a tissue contraction stage and a fibronectin scaffolding stage (Fig. 5c). The deposition of new scaffolding was further characterized by tangentially moving fibroblasts at the wound edge that assembled fibronectin into fibrillar networks within the gap, thus providing a provisional substrate for cell entry into the gap area. Once the opposing gap edges were close to each other, fibroblasts were able to span the gap and reinforce the region by thickening the tissue in the vertical plane (Fig. 5d). Interestingly, a similar closure mechanism involving ECM remodelling has been observed during eyelid closure in embryonic development. In this process, epidermal cells at the eyelid border pull on their surrounding ECM and intercalate perpendicular to the closure axis, a process dependent on α5β1-integrin/fibronectin interactions^6^. In agreement with these findings, we observed a similar dependence of gap closure on fibronectin-binding integrins and FAK signalling, although here intercalation did not occur. The fact that our model appeared to capture a distinct wound contraction phase and a deposition of provisional new matrix, to complete healing, further highlights the role of distinct cell–ECM processes to control this morphogenetic process.

Actomyosin contractility provides a central driving force for mediating many of the major structural reorganizations during morphogenesis^2^. Here, by establishing an approach to measure such forces during the repair of gaps, we demonstrated a staged process comprising relaxation after damage, followed by tissue contraction and a steady-state sub-baseline tension stage. Whereas rapid collagen contraction has been described in fibroblast-populated collagen lattices^30^, the demonstration of a relaxation phase and matrix deposition stage on either end of this process may provide a more complete picture of wound healing. Although such tension profiles have not yet been investigated *in vivo* after injury, the presence of such dynamics could regulate the wound-healing process at multiple levels. For example, transient loss of contractility has been described to stimulate increased motility^31^, and increased myosin activity is not only critical for ECM and tissue contraction, but also important for the assembly of fibronectin matrix^32^,^33^,^34^. Thus, there are several links between cytoskeletal forces and the many ensuing cellular remodelling events engaged during wound repair, and additional approaches to investigate and deconvolve these links are needed.

In adult skin, the tissue movements of wound repair involve re-epithelialization and fibrous tissue contraction^10^. Hence, bringing the wound margins together is a collective effort of epithelial cells and fibroblasts. Experiments with animal and 3D organotypic models revealed two distinct mechanisms for the repair of the dermis^35^,^36^. In a first mechanism, myofibroblasts contract the central granulation tissue in the wound to bring the wound margins closer^37^. Alternatively, fibroblasts residing at the wound edge can pull the intact dermis inwards by directional mass migration towards the centre of the wound^38^. In this latter mechanism, granulation tissue is not required for closure. Similarly, in our *in vitro* model, gaps spontaneously closed in the absence of a granulation tissue. The mechanical conditions and cellular origin that require the formation of a granulation tissue for the closure of full thickness wounds is unknown. A bioengineered model, such as ours, which allows one to controllably re-introduce the complexity of a wound environment, could provide a critical new strategy to help parse out the role of different cell types, matrix components, contractility and tissue remodelling in dermal tissue repair.

In summary, this study provides a new model to examine how cells are able to fill a void in free space, and demonstrates that stromal cells can close a tissue gap through the coordinated action of matrix contraction, cell migration and ECM remodelling. Importantly, morphogenetic events such as described herein involve spatial reorganization and deformation of ECM that cannot be captured by planar substrates. Hence, this bioengineered 3D model of wound closure may become a valuable tool to investigate the underlying mechanics of gap closure and ECM remodeling.

## Methods

### Device fabrication

Devices were fabricated as described previously^19^. Briefly, layers of SU-8 photoresist (Microchem) were patterned onto silicon wafers by successive spin coat, alignment, ultraviolet exposure and baking steps. All masters were developed in a single step in propylene glycol mehyl ether acetate (Sigma) followed by hard bake. Polydimethylsiloxane (PDMS, Sylgard 184, Dow-Corning) microtissue substrates were moulded from the SU-8 masters. Before cell seeding, the PDMS templates were sterilized in 70% ethanol followed by ultraviolet sterilization for 15 min before treatment with 0.02% pluronics-F127 (Sigma) solution for 10 min at room temperature.

### Cell culture

NIH 3T3 cells (American Type Cell Culture) were expanded in high-glucose (4.5 g l^−1^) DMEM containing (GIBCO) 10% bovine serum and 100 U ml^−1^ Penicillin and 100 mg ml^−1^ Streptomycin. Passage 4 to Passage 18 cells were used in our experiments.

### Microtissue formation and wound repair model

One million 3T3 cells were suspended in 2 mg ml^−1^ liquid neutralized collagen type I from rat tail (BD Biosciences) and seeded in the device. The entire assembly was centrifuged to drive cells into the chambers. Excess solution was removed, leaving solution only within the chambers, and the remaining constructs were centrifuged once again in an inverted configuration to resuspend the cells into the collagen matrix before polymerization. A few hours after polymerization, we observed the spontaneous contraction of the collagen matrix by the cells. Cantilevers incorporated within each chamber spatially restricted the contraction of the collagen matrix, whereby the contracting gels slide up the cantilevers and are then caught by the larger end caps, resulting in a large array of microtissues anchored to the tips of the cantilevers (Supplementary Fig. 1). To visualize the collagen and fibronectin matrix, 4% (w/w) Alexa-568-conjugated collagen (Alexa Fluor 568 labelling dye from ThermoFisher, A-20003) was mixed with the unlabelled collagen and Alexa-488-conjugated fibronectin (from human plasma, 8 μg ml^−1^, Alexa 488 labelling dye from ThermoFisher, A-20,000) was added to the medium during microtissue formation. After overnight incubation at 37 °C, cells contracted the collagen matrix around the engineered cantilever pillars and formed microtissues. Full-thickness incisional wounds were generated using a diamond dissecting knife (type MDL, Electron Microscopy Systems, #72029) mounted on an XYZ micromanipulator (SLC-2040, SmarAct GmbH) through a 3D printed plastic arm. The microtissues were cut layer-by-layer by teleoperating the arm at increasing depths using the visual feedback from the microscope as a guide.

### Inhibitor experiments

Medium containing pharmacological inhibitors was added just before damaging the microtissues. In this study we used RhoActivator II (10 ng ml^−1^, Cytoskeleton), RhoInhibitor (100 ng ml^−1^ Cytoskeleton), Y27632 (25 μM, Tocris), Blebbistatin (20 μM, Sigma), CytoD (4 μM, Sigma) and PF573.228 (5 μM, Tocris). For the antibody-blocking experiments, we added rat anti-mouse α5 (20 μg ml^−1^, clone 5H10–27 (MHR5), Abcam) and ArH anti-mouse β3 (20 μg ml^−1^, Clone 2C9.G3, Abcam). Rat IgG2a (20 μg ml^−1^, Abcam, ab18450) was used as isotype control.

### Two-dimensional elliptical scratch assay

PDMS stencils with four 500-μm-tall cylindrical pillars were microfabricated, treated with 0.2% F127 for 10 min and placed in a 12-well plate before seeding with 3T3 fibroblasts in growth medium. After the cells formed confluent monolayers, the pillars were removed and closure was evaluated in the presence of cytoskeletal drugs using time-lapse microscopy.

### Time-lapse microscopy

For wide-field imaging, microtissues were labelled with Hoechst 33342 (Sigma), to visualize the nuclei of the cells. Phase-contrast and fluorescent images were captured every 30 min for 24 h with a Photometrics Evolve 16-bit electron-multiplying charge-coupled device camera (Photometrics) and an A-Plan × 10 objective mounted on a Nikon Ti Eclipse (Nikon Instruments, Inc.) microscope equipped with a live cell incubator.

To visualize fibronectin dynamics, Alexa-488-conjugated fibronectin (from human plasma, 8 μg ml^−1^) was added to the medium during microtissue formation. The next day, medium was replaced before damaging. Twelve hours after wounding, samples were imaged with an LD-C apochromat 63 × 1.15 numerical aperture, water-immersion objective mounted to a Zeiss Axiovert 200M inverted microscope (Carl Zeiss) equipped with a CSU10 spinning disk confocal scan head (Yokogawa Electric Corp.) and live cell incubator. Time-lapse data were acquired every 20 min and 3-μm spacing in the axial plane. To reduce photoxicity, Oxyfluor (Fisher) was added to the medium (1:100). After image acquisition, the hyperstack was processed in imageJ using noise reduction filters, log transformation of the histogram, maximal *z*-projection and contrast/brightness enhancement. To maximize contrast of the fibronectin fibres in Fig. 4c, the histogram of maximal *z*-projections of image stacks taken at three different time points was inverted and recoloured using Photoshop CS4 (Adobe).

### Image processing and contractility measurements

Algorithms to measure the wound area and the size of the tissues from time-lapse videos were implemented in Matlab (Mathworks, MA). The programme accepts an input video and based on a brightness threshold generates regions that fill empty spaces inside and around the microtissues. These areas are analysed to calculate gap area, tissue width, gap shape and tissue shape. TrackMate, a plugin for ImageJ, was used to automatically track individual nucleus and characterize their trajectories from time-lapse videos. The tracks were then imported into Matlab and analysed for direction of motion and velocity. Windrose graphs were generated from histograms of several data points at different locations within the microtissues.

Contractility measurements were performed as described in our previous work^39^,^40^. Fluorescent microbeads (Fluoresbrite 17147; Polysciences, Inc.), embedded in the caps of the cantilevers, were used for computerized deflection tracking using the Spot Tracker plug-in for Fiji^41^. Briefly, the position of fluorescent beads located at the top surface of the cantilevers was measured during the course of the experiment. After recording the time-lapse data, tissues were disintegrated with collagenase, to determine the baseline position of the same beads. Cantilever displacements were measured by subtracting the baseline position of fluorescent beads from the position at a given time point. Total contractility was calculated by multiplying the sum of cantilever displacement with the spring constant (*k*=2.67±0.31 μN μm^−1^) of the cantilevers at a PDMS–curing agents ratio of 1:10. The spring constant of cantilevers was measured using capacitive force sensors (FT-S100, FemtoTools GmbH) mounted on a microrobotic manipulation platform^42^.

### Immunohistochemistry

Microtissues were fixed with 4% paraformaldehyde in PBS, permeabilized with 0.2% Triton X-100 in PBS, blocked in 10% Goat serum for 1 h at room temperature, followed by incubation with antibodies against phospho-myosin light chain 2 (Thr19/Ser19) (Cell Signaling Technologies, #3674L, 1:100), fibronectin (Abcam, ab2413, 1:100), cellular fibronectin (Sigma, clone FN-3E2, #F6140, 1:100) and N-Cadherin (Cell Signaling, clone 13A9, #14215S, 1:100) overnight at 4 °C, and detected with goat anti-rabbit Alexa 568 (1:1,000)-conjugated antibodies.

### N-Cadherin knockdown experiments

3T3 fibroblasts in p60 petri dishes were transfected with either scrambled siRNA (AllStars Negative Control siRNA, 50 μM) or a pool of four siRNAs targeting Cdh2 (FlexiTube GeneSolution, GS12558, 50 μM) using HiPerfect Transfection Reagent (all from Qiagen). Two days after transfection, transfected cells were seeded in microtissues, which were wounded the next day. The remainder of the cells was re-plated on fibronectin-coated PDMS coverslips to assess N-cadherin expression with immunohistochemistry.

### Statistical analysis

Results are presented as mean±s.e.m. Statistical analysis was performed using JMP Pro 11 (SAS Institute). Differences between experimental conditions were compared by analysis of variance followed by *post-hoc* Dunnett's test. For the migration data, a Kruskal–Wallis test followed by a Dunn Method For Joint Ranking *post-hoc* analysis was used. Significance values are indicated as *P≤0.05, **P≤0.01 and ***P≤0.001.

### Code availability

Please see Supplementary Data 1 and 2 to access the computer code generated for processing the time-lapse images of microtissues and quantifying tissue morphology.

## Additional information

**How to cite this article:** Sakar, M. S. *et al*. Cellular forces and matrix assembly coordinate fibrous tissue repair. *Nat. Commun.* 7:11036 doi: 10.1038/ncomms11036 (2016).

## Supplementary Material

Supplementary Figures Supplementary Figures 1-5

Supplementary Data 1 The computer code 1 generated for processing the time-lapse images of microtissues and quantifying tissue morphology.

Supplementary Data 2 The computer code 2 generated for processing the time-lapse images of microtissues and quantifying tissue morphology.

Supplementary Movie 1 The progressive closure of gaps over the course of 24 hours after injury. Microscopy images were taken every 30 minutes.

Supplementary Movie 2 The progressive closure of gaps over the course of 24 hours after injury in the presence of Aphidicolin (30 μM), a mitogenic inhibitor. Microscopy images were taken every 30 minutes.

Supplementary Movie 3 Time-lapse microscopy of gap closure by 3T3 fibroblasts on 2D substrates and in 3D microtissues.

Supplementary Movie 4 Cell migration around the wound area. Microtissues were labeled with Hoechst 33342 to visualize the nuclei of the cells. Phase contrast and fluorescent images were captured every 30 minutes for 24 hours.

Supplementary Movie 5 Time-lapse microscopy of gap closure in microtissues containing fluorescently tagged collagen type I matrix. Fluorescent images were captured every 30 minutes for 24 hours.

Supplementary Movie 6 Live imaging of fibronectin dynamics during gap closure. Alexa-488 conjugated fibronectin (8μg/ml) was added to the medium during microtissue formation. Images were acquired every 20 minutes and 3-μm spacing in the axial plane.
