# Supplementary material for: Cellular forces and matrix assembly coordinate fibrous tissue repair
Source: Nat Commun. 2016 Mar 16;7:11036. doi: 10.1038/ncomms11036 (PMC4799373; doi:10.1038/ncomms11036)
Supplement: Supplementary Figures — 1-5 [file ncomms11036-s1.pdf]

Supplementary Figures

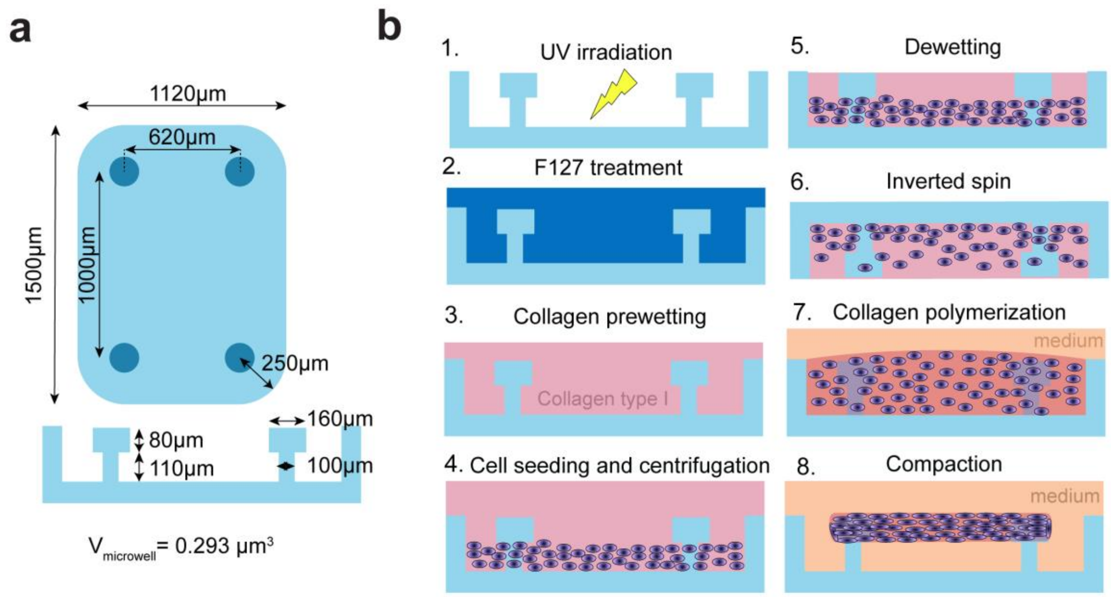

Supplementary Figure 1 Experimental design for engineering 3D stromal microtissues using microfabricated tissue gauge technology. (**a**) Schematic view of a single chamber showing dimensions of individual components. (**b**) Process flow diagram for the formation of microtissues. The molds are first UV sterilized (1) and treated with a surfactant (2) to minimize adverse adhesion. The mold was then immersed in a cooled suspension of cell laden neutralized collagen (one million cells) and the entire assembly was centrifuged (4) to drive cells into the chambers. Excess collagen solution was removed (5) and the remaining constructs were spun again in an inverted configuration (6) to move the cells closer to the caps of the cantilevers. A few hours after polymerization (7), cells spontaneously contracted the collagen matrix (8). Cantilevers incorporated within each chamber spatially restricted the contraction of the collagen matrix, which resulted in a large array of microtissues anchored to the tips of the cantilevers. Each tissue contained approximately 2000 cells.

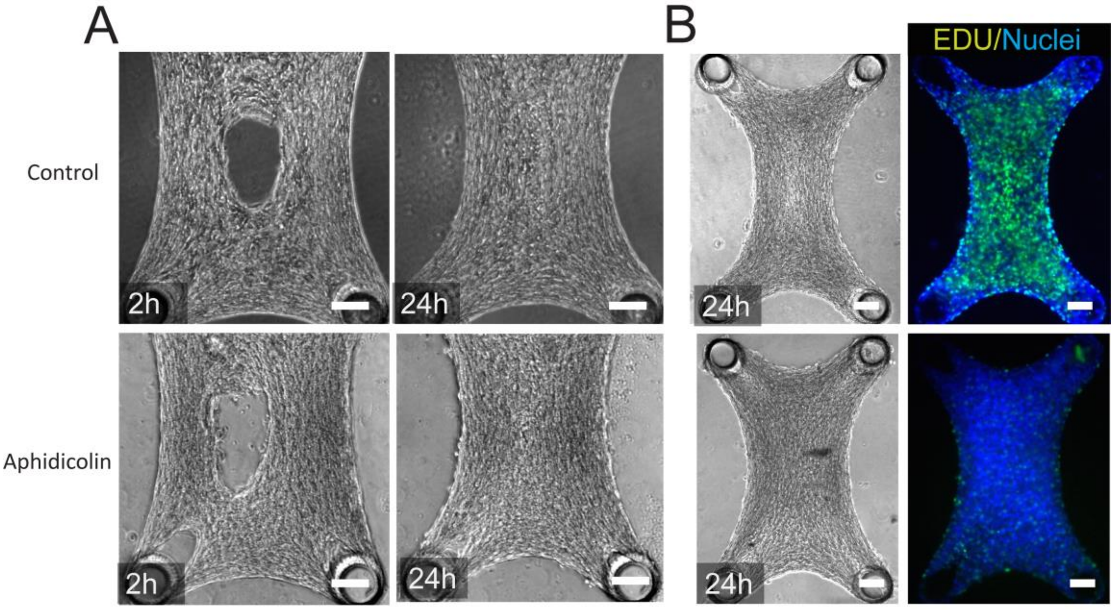

Supplementary Figure 2. Proliferation has limited impact on gap closure. Microtissues were treated with 10 µg/ml of Aphidicolin show less EDU incorporation during healing compared to DMSO treated control tissues and can close within 24h. Scale bar: 100µm.

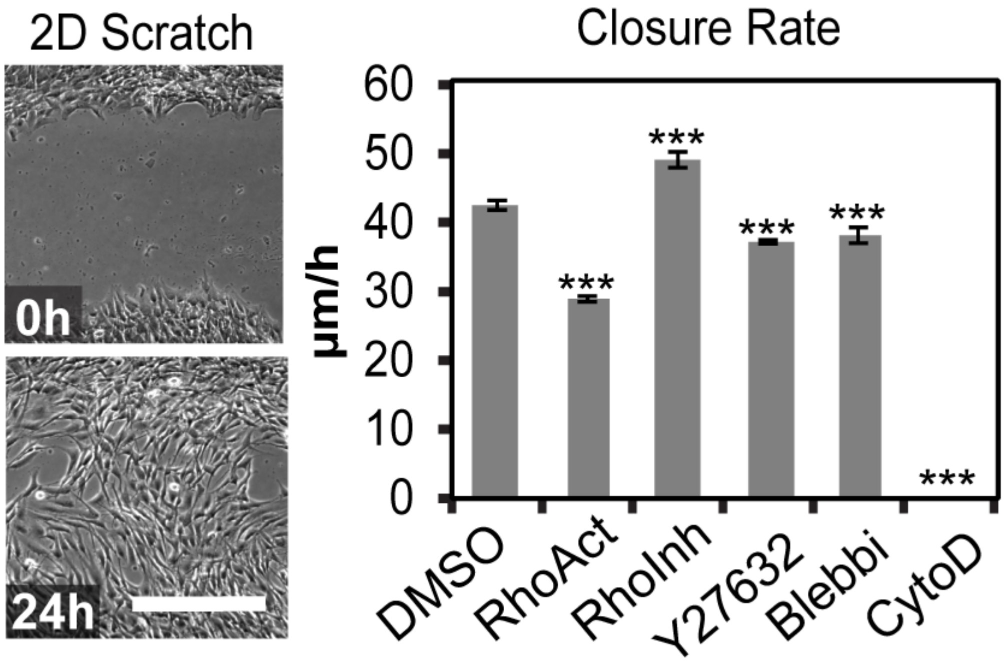

Supplementary Figure 3 Classical parallel scratch wound assay. Closure rate of 2D scratch wounds in presence of cytoskeletal drugs (N= 3, 3 experiments). All bar graphs represent average ±SEM. Statistical analysis: ANOVA, post-hoc Dunnett's for comparison to the DMSO control, $^{*}P \leq 0.05$, $^{**}P \leq 0.01$, *** $p<0.001$. Scale bar: 100µm.

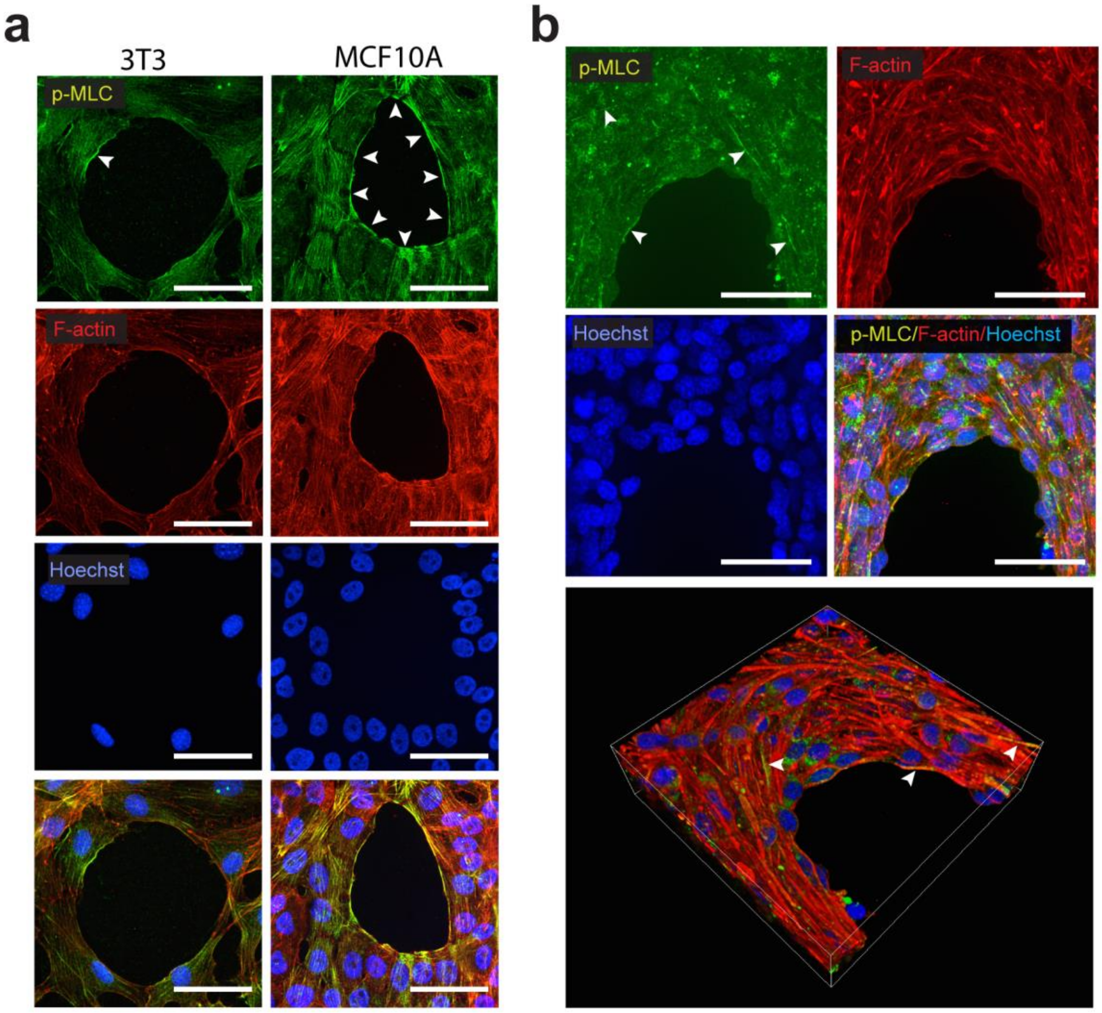

Supplementary Figure 4. Fibroblasts do not employ an actomyosin purse-string to close a gap. (**a**) Immunohistochemical staining of monolayers of fibroblast (3T3) and epithelial cells (MCF10A) on collagen type I substrate for phosphorylated Myosin Light Chain (phospho-MLC, green), counterstained with Phalloidin (red) and Hoechst (blue) (Magnification 40x). Removing PDMS pillars surrounded by cells created elliptical gaps. Phospho-MLC consistently accumulated at the gap margin in epithelial monolayers (white arrowheads) but not in fibroblasts. (**b**) Confocal micrographs (maximum z-projection) and 3D reconstruction of confocal slices show that phospho-MLC is sporadically present in single fibroblasts (white arrowheads), but is not accumulated at the wound edge to form a purse-string during the closure of gaps in 3D stromal microtissues. Scale bars: 50µm.

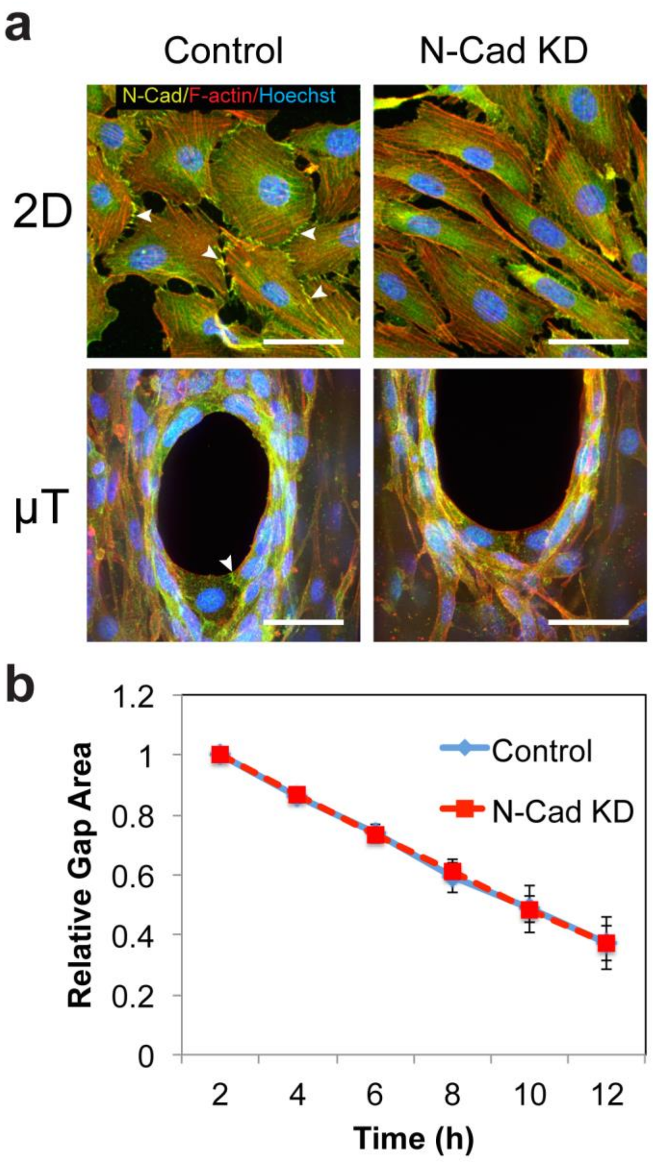

Supplementary Figure 5. Knock down of N-cadherin does not interfere with gap closure. N-cadherin expression is silenced using a pool of four siRNAs targeting Cdh2. Scrambled siRNAs and scrambled siRNA fluorescently labeled with Cy3 were used as controls. (**a**) Staining for N-cadherin in the control condition showing N-cadherin expression in 3T3 fibroblasts in monolayer (2D) and during closure in microtissues (µT) (N-Cad: green, F-actin: red, Nuclei, blue, magnification 40x). When treated with siRNA targeting Cdh2, N-Cadherin expression was knocked down (N-Cad KD). (**b**) Microtissues generated from 3T3 treated with siRNA targeting Cdh2 closed gaps with the same speed as the control tissues. Scale bars: 50µm.
